# To Compare the Maternal and Fetal Outcomes of COVID-19-Affected Expectant Mothers During the First and Second COVID-19 Waves: Data From a Tertiary Care Referral Hospital in Punjab

**DOI:** 10.7759/cureus.36319

**Published:** 2023-03-17

**Authors:** Isha Tapasvi, Amanpreet Sethi, Chaitanya Tapasvi, Seema Grover, Parveen Rajora

**Affiliations:** 1 Obstetrics and Gynecology, Guru Gobind Singh Medical College and Hospital, Faridkot, IND; 2 Pediatrics, Guru Gobind Singh Medical College and Hospital, Faridkot, IND; 3 Radiology, Guru Gobind Singh Medical College and Hospital, Faridkot, IND

**Keywords:** first and second waves, antenatal complications and comorbidities, neonatal outcome, maternal outcome, covid-19 infection

## Abstract

Background: Coronavirus 2019 (COVID-19) infection, declared pandemic in March 2020 by the World Health Organization, paved the way for newer research in the field of medicine. The second wave, beginning in March 2021, appeared to be more devastating. The purpose of this study is to evaluate the clinical characteristics, effects of COVID-19 infection in pregnancy, and obstetric and perinatal outcomes in the first and second waves.

Materials and methods: This study was conducted from January 2020 to August 2021 at the Guru Gobind Singh Medical College and Hospital, Faridkot, Punjab. The patients were enrolled immediately after each infected woman was identified as per the inclusion and exclusion criteria. Demographic details of the patients, associated comorbid conditions, intensive care unit (ICU) admission, and treatment details were noted. Neonatal outcomes were recorded. The testing of pregnant women was done as per the Indian Council of Medical Research (ICMR) guidelines.

Results: There were 3421 obstetric admissions and 2132 deliveries during this period. Group 1 had 123 COVID-19-positive admissions, while group 2 had 101 admissions. The incidence of COVID-19 infection in pregnancy was 6.54%. In both groups, the majority of patients were between the ages of 21 and 30. About 80(66%) of the admissions in group 1 and 46(46%) in group 2 were in the gestational age of 29-36 weeks. Multiparity was more common in both groups, with 58% of cases in group 1 and 79% of cases in group 2. Obstetric comorbidities were common in both groups, seen in 46% of cases in group 1 and 78% of cases in group 2. The majority of patients were asymptomatic in group 1, with an 89% incidence, whereas only 33% of patients in group 2 were without symptoms.

In biological data, D-dimers, prothrombin time, and platelet count were altered in 11%, 14%, and 17% of cases, respectively, in group 2, with almost normal data in group 1. Most cases in group 2 (52%) were critical cases in the moderate and severe categories requiring intensive care unit (ICU) treatment, whereas there was only single ICU admission in group 1. The overall case fatality rate (CFR) in group 2 was found to be 19.8(20/101).

Delivery by cesarean section was done in 38.2% of cases in group 1, while in 33% of cases in group 2, with a significant p-value of 0.001. About 29% of cases in group 1 and 34% of cases in group 2 underwent vaginal delivery. The rate of abortion was almost similar in both groups. Only two cases in group 1 and nine cases in group 2 had intrauterine fetal death. Observations of neonatal outcomes suggested that five cases in group 2 and two cases in group 1 had severe birth asphyxia. Only one case in group 1 and four cases in group 2 had positive COVID-19 status.

Maternal mortality was significantly higher in group 2 with 20 cases, while only one case was in group 1. Anemia and pregnancy-induced hypertension were the chief comorbidities in this group.

Conclusion: COVID‐19 infection during pregnancy may be associated with maternal mortality while having a minimal effect on neonatal morbidity and mortality. The possibility of maternal‐fetal transmission cannot be ruled out completely. The severity and characteristics of COVID-19 may vary in each wave, and we need to modify treatment strategies. More studies or meta-analyses reports are required to authenticate this transmission.

## Introduction

Coronavirus disease 2019 (COVID-19) infection declared a pandemic in March 2020 by the World Health Organization [1] paved the way for research and a newer outlook in the field of medicine. In its journey from Wuhan, China in December 2019 to the world till date and with various waves, it has reached millions, sparing no age, no gender, and no class of population. Pregnancy, earlier considered an immunocompromised state [2], is rather a state of immune modulation and saw its worst time during COVID-19, raising the bars of maternal and fetal mortality [3,4].^ ^

The second wave, beginning in March 2021, was more devastating than the first, with shortages of vaccines, hospital beds, oxygen cylinders, and other medical supplies in various parts of the country [5]. Wave 1 was from January 2020 to January 2021, with a peak in mid-September, and wave 2 was from March 2021 to August 2021 [5]. In one of the preliminary studies of 141 patients during the early pandemic, it was concluded that there was no significant effect of COVID infection on maternal and fetal outcomes in pregnancy [6]. The study aimed to evaluate the clinical characteristics and effects of COVID-19 infection in pregnancy in the first and second waves as well as obstetric and perinatal outcomes highlighting the possible causes for the differences in the same.

## Materials and methods

Design, setting, and participants 

We conducted a prospective, longitudinal, observational study of all COVID-positive antenatal and postnatal women admitted between January 2020 and August 2021 in the Department of Obstetrics and Gynecology and the Neonatology Unit of the Pediatrics Department at Guru Gobind Singh Medical College and Hospital, Faridkot. Patients were enrolled immediately after each infected woman was identified, at any stage of pregnancy or delivery, as per the inclusion and exclusion criteria (Table 1).

**Table 1:** Inclusion and exclusion criteria. COVID-19: coronavirus disease 2019.

Inclusion criteria
Hospitalized COVID-19-positive antenatal cases.
Post-partum cases referred due to COVID-19-positive status.
Neonates of COVID-19-positive mother.
Exclusion criteria
Suspected COVID-19 infection without laboratory confirmation.
Patients who refuse consent.

Maternal information, including demographic data, disease history during the antenatal period, time of onset of disease, type of clinical symptoms, associated comorbidities, and mode of delivery, was noted. In the exposed neonates, data regarding gestational age at birth, birth weight, mode of delivery, and signs of sepsis were collected. Inborn neonate reverse transcriptase-polymerase chain reaction (RT-PCR) samples were sent immediately after delivery. This study was approved by the Guru Gobind Singh Medical College and Hospital Institutional Ethics Committee with registration number ECR/836/Inst./PB/2016/RR-20, GGS/IEC/72.

Methodology

Demographic details of the patients, like age, address, parity, period of gestation and clinical symptoms, associated comorbid conditions and treatments taken, were noted. Neonatal outcomes, including appearance, pulse, grimace, activity, and respiration (APGAR) score, were recorded.

We collected and compared data on (1) obstetric complications like preterm labor, antepartum hemorrhage, fetal distress, preterm rupture of membranes, intrauterine growth restriction, and maternal mortality in confirmed COVID-19-positive pregnant women. (2) Intrauterine fetal death cases with no identifiable obstetric cause. (3) Neonatal complications like perinatal asphyxia.

The testing of pregnant women was done as per Indian Council of Medical Research (ICMR) guidelines, which stated that pregnant women residing in containment zones or from hotspot districts presenting in labor or likely to deliver in the next five days should be tested even if asymptomatic. Patients were managed as per guidelines in separate COVID wards under obstetrician and physician care. For neonates delivered to a COVID-positive mother, nasal swabs were sent 24 hours after birth as per institutional protocol, and results were noted.

Sample size

All the eligible patients and a convenient sample size of 224 patients were enrolled.

Statistical analysis

Data were described in terms of range; mean±standard deviation (±SD), frequencies (number of cases), and relative frequencies (percentages) as appropriate. To determine whether the data were normally distributed, a Kolmogorov-Smirnov test was used. The comparison of quantitative variables between the study groups was done using the Mann-Whitney U test for independent samples for non-parametric data. For comparing categorical data, the chi-square (χ^2^) test was performed, and the Fisher exact test was used when the expected frequency was less than 5. A probability value (p-value) less than 0.05 was considered statistically significant. All statistical calculations were done using the Statistical Package for the Social Sciences (SPSS) 21 version (SPSS Inc., Chicago, IL, USA) statistical program for Microsoft Windows.

Definitions

Date of diagnosis: It is derived from the earliest date of either (1) when the reverse transcriptase-polymerase chain reaction (RT-PCR)/cartridge-based nucleic acid amplification test (CBNAAT) report was received or (2) the earliest available date related to the illness or specimen collection.

COVID-19-associated maternal death: A COVID-19-associated death after severe acute respiratory syndrome coronavirus 2 (SARS-CoV-2) infection during pregnancy was defined as the death of a woman with confirmed or probable SARS-CoV-2 infection during pregnancy who subsequently died during pregnancy or within 90 days after the pregnancy ended.

Case fatality rate (CFR): It is the proportion of people diagnosed with a certain disease who end up dying of it.

COVID-19 exposure in pregnancy: Exposure to COVID-19 in pregnancy is determined by laboratory confirmation of COVID-19 and/or radiological pulmonary findings anytime during treatment or admission or by two or more predefined COVID-19 symptoms.

Vertical transmission: It is the passage of a disease-causing agent (pathogen) from mother to baby during the period immediately before and after birth.

## Results

During the study periods of both waves, there were a total of 3421 obstetric admissions and 2132 deliveries. Of these, wave 1 (group 1) had 123 COVID admissions with 85 deliveries, and wave 2 (group 2) had 101 admissions with 73 deliveries. So, the incidence of COVID infection in pregnancy in our institute was 6.54.

On observing the demographic profile (Table 2) of the patients, the majority of the patients were found in the 21-30 years age group. Wave 2 had a significant variation with relatively young cases than wave 1, with a p-value of 0.003. Both waves had a higher number of multigravida women than primigravida women. Thus, no significant variation was identified. Maximum cases (66%) in group 1 and 46% in group 2 were in the 29-36 week period of gestation, with a significant variation of 0.019.

**Table 2:** Demographic profile.

Variable	Group 1 cases, n=123	Group 2 cases, n=101	p-value
Demographics			
Age			
<20 years	12(10%)	8(8%)	0.03
21-30 years	67(55%)	78(77%)	0.03
31-40 years	40(33%)	15(51%)	0.03
>40 years	3(2%)	0(0%)	0.03
Gestational age			
<12 weeks	3(2%)	3(3%)	0.019
13-28 weeks	10(8%)	18(18%)	0.019
29-36 weeks	80(66%)	46(46%)	0.019
>36 weeks	29(24%)	34(34%)	0.019
Parity			
Primigravida	43(35%)	43(43%)	
Multigravida	79(65%)	58(58%)	

In Table 3, the main co-morbidities in both groups were obstetrical 46% in group 1 and 78% in group 2. The incidence of medical illness was almost double in group 2.

**Table 3:** Main comorbidities.

Main comorbidities	Group 1	Group 2
Medical	10(8%)	20(20%)
Endocrine	1	5 (5%)
Coagulation	13(10%)	16(16%)
Obstetrical	56(46%)	78(78%)

Table 4 identifies the common obstetric comorbidities. These were the high-risk pregnancy cases treated in both groups. Anemia (16%) and pregnancy-induced hypertension (19%) were the most common comorbidities in group 2. The incidence of amniotic fluid disorders (12%) was slightly higher in group 2, while the incidence of premature rupture of membrane (13%), antepartum hemorrhage (8%), and intrahepatic cholestasis of pregnancy (2%) was similar in both groups.

**Table 4:** Obstetric comorbidities.

Comorbidities	Group 1, n=123 (%)	Group 2, n=101 (%)
Pregnancy-induced hypertension	11	19
Multiple pregnancies	2	2
Amniotic fluid disorders	8	12
Premature rupture of membrane	11	13
Antepartum hemorrhage	6	8
Intrahepatic cholestasis of pregnancy	3	2
Others (anemia)	13	16

Table 5 shows that most of the patients in group 1 were asymptomatic or had mild symptoms in the form of cough, dyspnea, or fever and did not require intensive care, while in wave 2, the majority of the patients were symptomatic. Fever was seen in 38% of cases.

**Table 5:** Clinical profile.

	Wave 1 (n=123)		Wave 2 (n=101)		Total	p-value
	No. of cases	% Age	No. of cases	% Age		
Asymptomatic	108	89%	33	33%	141	0.001
Sore throat	0	0%	4	4%	4	0.027
Cough	6	5%	22	22%	28	0.001
Dyspnea	4	3%	20	20%	24	0.001
Fever	6	5%	38	38%	44	0.001
Desaturation	0	0%	10	10%	10	0.001
Diarrhea	0	0%	2	2%	2	0.118
Others	0	0%	6	6%	6	0.006

Table 6 describes that the biological data was more altered in group 2 rather than group 1, with D-dimers, prothrombin time, and platelet count altered in 11%, 14%, and 17% cases, respectively.

**Table 6:** Biological data and ICU scores. HDU: high dependency unit, ICU: intensive care unit.

	Group 1	Group 2
D-dimer (ng/ml)	2	11
Prothrombin time (%)	1	14
Fibrinogen (g/L)	2	9
Platelets count	5	17
Treatment administered at ICU		
Inotropic support	-	16
Anti-coagulation	-	15
Antibiotic therapy for bacterial co-infection at ICU admission	-	21
Outcome at ICU/HDU		
ICU admissions	-	21
Invasive mechanical ventilation (IMV)	-	20
Nasal cannula	-	3
Non-rebreather mask	-	5
Non-invasive ventilation (NIV)	-	23
HDU admissions	1	52
Discharged	-	33
Mortality	1	20
HDU survival rate	-	57%
Main delay in treatment		
<7 Days between disease onset and hospital/ICU admission	101	44
>7 Days between disease onset and hospital/ICU admission	22	57

Most cases in group 2 (52%) were in the moderate and severe categories of COVID infection, which were managed in the high dependency unit (HDU)/intensive care unit (ICU) as they were sick admissions. Of the 21 cases of ICU admissions during wave 2, 16 cases (76%) required inotropic support, 19 cases (90%) required ventilatory support, and 15 cases (71%) required anti-coagulation. The average duration of the ICU stay in wave 2 was 5-10 days. However, in one case, the stay was prolonged to 22 days, and the patient was discharged in stable condition after treatment. There were 20 ICU mortalities in group 2. Out of these, injection remdesivir was given to three patients, but the outcome could not be improved. It was observed that in group 1, there was not much delay in taking treatment, with the maximum number of cases 101(82%) admitted after diagnosis in <7 days interval period, whereas in group 2, only 44(44%) sought early treatment and 57(57%) cases were admitted after seven days interval.

In Table 7, the number of patients delivered by lower segment cesarean section (LSCS) in group 1 (38.2%) were higher as compared to group 2 (33%), with a significant p-value of <0.05. Observation of the neo-natal outcome shows that both groups had normal APGAR scores, with an insignificant p-value. Birthweight comparison showed similar results with maximum cases in the 2-3 kgs group with an insignificant p-value of 0.959. In group 1, four cases (3%) and three cases (3%) in group 2 had abortions in the first trimester. About 26(21%) cases in group 1 and 22 cases (22%) in group 2 were managed conservatively with a significant p-value and were discharged with follow-up advice. In group 1, only one neonate came out to be COVID-19 positive, while in the second group, four neonates acquired COVID-19 infection.

**Table 7:** Delivery data with neonatal outcome. NVD: normal vaginal delivery, LSCS: lower segment cesarean section, IUD: intrauterine death, Apgar score: appearance, pulse, grimace, activity, and respiration, RT-PCR: reverse transcriptase-polymerase chain reaction.

Mode of delivery	Wave 1, n=123	Wave 2, n=101	p-value
NVD	36	34	0.001
LSCS	47	30	
IUD	2	9	
Abortions	4	3	
Conservative	26	22	
Neonate outcome	Wave 1	Wave 2	
Apgar score			
No asphyxia	75	57	0.625
Moderate asphyxia	5	2	0.404
Severe asphyxia	2	5	0.123
Total	83	64	
COVID-positive (RT-PCR) neonate	1	4	
Birth weight (kg)			
>3	11	6	0.466
2-3	69	53	0.959
1-2	3	5	0.266
<1	-	-	-

Table 8 shows the rate of maternal death in COVID-19 patients was significantly higher in group 2 than in the other group. Twenty cases (19.8%) in group 2 had mortality compared to only one mortality out of 123 admissions in group 1 (p<0.05). The only mortality in group 1 was a primigravida with 37 weeks of gestation with eclampsia and deranged liver function tests. All the severe disease cases in group 2 were immediately shifted to the ICU. Most of the patients, 10 out of 20(50%) were in critical condition in HDU/ICU for 48-72 hours, while four out of 20(20%) succumbed to death within 24-48 hours. In wave 1, home isolation was opted by 93% of cases, whereas in wave 2, it was opted by 78% of cases with a significant p-value of 0.001.

**Table 8:** Maternal mortality data. GDM: gestational diabetes mellitus, PIH: pregnancy-induced hypertension.

Gestational age (weeks)	Wave 1 (n=1)	Wave 2 (n=20)
<28	-	4
29-36	-	9
>37	1	
Postpartum	-	3
Total	1	20
Comorbidities	Wave 1	Wave 2
GDM	-	3
PIH/eclampsia	1	6
Anemia	-	9
Cardiac disease	-	3
Respiratory disease	-	5
Time interval between admission and death	Wave 1	Wave 2
<24 hours	1	3
24-48 hours	-	4
48-72 hours	-	10
72 hours	-	3

 In our study, the overall case fatality rate (CFR) in group 2 with COVID-19 infection was 19.8(20/101).

## Discussion

This study was done to analyze the factors associated with the differences in maternal and fetal outcomes due to COVID-19 infection in the first and second waves. The majority of the patients were asymptomatic in both groups. However, the main presenting symptoms in group 2 were cough, dyspnea, and fever. Various studies [7-9] showed that these were the common presenting symptoms in pregnant and non-pregnant cases during COVID time.

Group 2 had higher medical and other comorbidities, the most common of which were anemia (8.9%) and pregnancy-induced hypertension (5.9%). In a study by Mullins et al. [10], pregnancy-induced hypertension, seen in 31.37% of cases, was the commonest comorbidity. In our study, most of the infections reported were in the 29-36 weeks period of gestation (time between conception and birth), similar to the study by Fan et al. [11], in which both cases reported were in the third trimester.

In various studies done by Chen et al. [12] and Yu et al. [13], it was reported that LSCS was done for all COVID-positive patients. In our study, LSCS was done only for obstetric indications and associated comorbidities like previous LSCS, meconium-stained liquor, and fetal distress.

Newborn characteristics showed that the majority of neonates were born with a normal Apgar index score (7-10) in both waves, with almost similar figures in neonatal intensive care unit (NICU) and nursery admissions.

Chen et al. [12] and Qiancheng et al. [14] have suggested that currently there is no evidence for intrauterine infection caused by vertical transmission in women who develop COVID-19 pneumonia in late pregnancy. However, our study reported five cases of vertical transmission, four cases in group 2 (6.25% infectivity) and only one in wave 1. This may be due to the geographic and ethnic variations of the study population and the probability of infection by different COVID-19 strains. These neonates were managed conservatively in the COVID nursery, and all recovered well except for one case, which succumbed to sepsis and pneumonia.

Data regarding maternal and neonatal outcomes due to the severity of COVID-19 infection in pregnancy is limited as the adverse outcomes are related to non-obstetric causes like viral pneumonia complicating pregnancy [15]. A study by Creanga et al. [16] highlighted the potential for severe illness and adverse neonatal outcomes among pregnant H1N1 influenza-infected women.

Our study included both mild and severe disease cases with acute respiratory distress syndrome (ARDS) in the first and second waves. Mild cases were managed with supportive treatment, while severe cases required ICU/HDU care in wave 2. This is in contrast to a study by Horby et al. [17], wherein patients had fewer ICU admissions. Qiancheng et al. [14] also concluded in their study that pregnant women had comparable clinical course and outcomes as with non-pregnant women with SARS-CoV-2 infection. The case fatality rate (CFR) in our study was 19.8% as compared with Gajbhiye et al. [18], wherein it was 0.8% (34/4203). Our study saw increased maternal mortality in group 2, which might have been due to a delay in referrals to higher centers due to the ongoing pandemic and the high severity of disease in wave 2. This difference may also be increased due to sick patient referrals at our tertiary care hospital at critical stages requiring ICU care with an increase in the severity of COVID-19 infection in group 2. Another reason for the differences in results between the waves may be explained by the increased virulence of COVID-19 strains, which led to the sudden exponential growth of COVID-positive cases in wave 2, leading to the saturation of healthcare facilities in our region. 

Limitations

This study is done in a small geographical area of North Punjab with a limited sample size. Limited data were available in India on maternal mortality throughout all the COVID-19 waves.

## Conclusions

Our study is an effort to impart more knowledge about the various outcomes of COVID-19 pregnancy for a better understanding of this disease. From our observations, COVID‐19 during pregnancy may be associated with severe maternal morbidity and maternal mortality, and the possibility of maternal‐fetal transmission cannot be ruled out completely at present. Larger studies or meta-analyses reports are required to authenticate this transmission. The results of our study indicate that the characteristics and severity of SARS-CoV-2 may vary with each COVID wave, and we need to modify treatment strategies to reduce maternal morbidity and mortality.
